# There is Room for Even More Doublethink: The Perilous Status of Psychoanalytic Research

**DOI:** 10.1080/10481885.2013.754276

**Published:** 2013-02-06

**Authors:** Peter Fonagy

**Affiliations:** University College London

## Abstract

The opposition between psychoanalysis and systematic interdisciplinary research is to be regretted. The target article attempts to bridge the intellectual divide and for this aim as well as the intellectual adroitness shown it is to be celebrated. Much harder to understand is the high level of affect generated by the debate. Accusations of “doublethink” are helpful. The present paper, like the target paper it follows, attempts to develop an understanding of the position of those who are categorically opposed to interdisciplinary systematic research linked to psychoanalysis. Appreciating the perspective of those deeply opposed to such work could help to create a shared agenda from which our troubled discipline could benefit. This is predicated on the possibility of an open collegial dialogue which this journal was founded to create.

I offer this contribution in appreciation and strong support of Jeremy Safran's (2012) critique of Irvin Hoffman's broadside against “Doublethinking” by psychoanalytic researchers. Amongst life's many ironies was the coinciding of the appearance in the *Journal of the American Psychoanalytic Association* of Irvin Hoffman's (2009) paper and the appearance in the *British Journal of Psychiatry* of a three-round debate between Lewis Wolpert, the renowned developmental biologist, and me with the theme “There Is No Place for the Psychoanalytic Case Report in the *British Journal of Psychiatry*” (Wolpert & Fonagy, 2009). I was arguing against Wolpert, who proposed that the psychoanalytic case study had *no* information of significance to contribute to modern psychiatry. To argue for case reports in the *British Journal of Psychiatry* was admittedly fairly hopeless, given that so few are published in this high impact journal. The debate was perhaps set up in this way by the editor of the journal, Professor Tyrer, who is no great fan of psychoanalytic psychiatry, perhaps to discourage psychoanalysts from submitting case reports to him (hardly a serious risk). But imagine my surprise when I returned bloodied from this debate and picked up the *Journal of the American Psychoanalytic Association*, “my home team's journal,” to find myself depicted as a dangerous opponent of case studies. I had been rhetorically tried and convicted of “scientific doublethink” in advocating that our discipline should join forces across psychoanalytic schools in an “effort to make the best case possible for psychoanalysis as a clinical method” (Fonagy, 2002, p. 58).

## THE STATUS OF THE RANDOMIZED CONTROLLED CLINICAL TRIAL

Once I had overcome my immediate reaction, which was a toxic combination of self-pity and indignation, I started reflecting on what might be the underlying issues here. I am gratified to see that Jeremy Safran arrived at the same conclusions with greater clarity and presented them with superior eloquence than I could then or now have mustered. I agree with Jeremy Safran that no one would get out of bed to defend randomized controlled clinical trials (RCTs). Not even the leader of evidence based medicine in the United Kingdom—Sir Michael Rawlins, the Director of our National Institute for Health and Clinical Excellence—sees RCT methodology as a royal road to understanding outcomes. In his Harveian Oration, Rawlins (2008) identified many major bioethical, legal, and conceptual problems with RCTs, including small sample sizes requiring massive effect sizes for significance; additional difficulties if the aim of the RCT was to show no difference (the problems of equivalence trials, nonsuperiority studies, futility designs); lack of generalizability of the results collected from selected populations for a finite time as opposed to real heterogeneous populations with comorbidities, usually treated over longer periods of time, from different ethnic backgrounds and socioeconomic status; differences in the dose, timing, and duration of therapy, comedication and comparative effectiveness, and so on. He asks the nontrivial question: Can the benefits for the average patient in an RCT be extrapolated to “average” patients in clinical settings? Now, is Sir Michael Rawlins, the person in charge of the organization that defines appropriate care for the 30 million patients seen in the British National Health Service each year, guilty of Orwellian doublethink for raising questions about the key tool that his organization uses to define protocols for all but the most unusual of disorders? I don't think so. He is showing skepticism appropriate to scientists about the limitations of his experimental methods.

The doublethink is slightly more pernicious in our case. Hoffman (2009, p. 1056, in a “telling footnote”) referred to a recent report by Leichsenring and Rabung (2008) concerning the effectiveness of long-term psychodynamic psychotherapy in terms of effect size, relative to the effects normally observed in short-term cognitive behavioral treatments. This citation, while of little significance in itself, illustrates a larger problem. Hoffman, and many other psychoanalysts, would readily accept uncritically the authors’ claim of a demonstration of superiority because it fits with our a priori views. In fact, the limited number of studies available (summarized in the *Journal of the American Medical Association* article) are not necessarily representative of ordinary psychoanalytic practice in terms of either the patient population that was under scrutiny (largely a severely personality disordered group) or the therapeutic procedures used in the reports summarized. These facts do not escape the scientist but might be overlooked by those who wish to search for and find evidence for the validity of their current practice. Many of us have argued that training in research methodology should be taken more seriously by psychoanalytic institutes in order to avoid this, and to ensure that such judgments are made in a more sophisticated way (Emde & Fonagy, 1997).

The purpose of undertaking systematic empirical studies (qualitative or quantitative) is *not* to confirm beliefs already held but to expose oneself to the possibility of surprise. Not that this is by any means the privileged domain of the empiricist. I was certainly surprised to read the extraordinary claim that clinical psychoanalytic research is free from risk of a confirmatory bias because the “ambiguity of psychoanalytic data leave them relatively unmanipulable in the sense of stacking the cards in favor of one or another point of view” (Hoffman, 2009, p. 1052). This is a strong empirical statement, and if confirmed would indeed place psychoanalysis in a privileged epistemic position. It is hard to see, given what psychoanalysis has taught us about the power of unconscious emotional control over human cognition, that the naivety of the average clinician would be such as to render him or her unable to bias her or his memory in the direction of greater compliance with convictions, even if these are only unconsciously held. Jeremy Safran accepts Hoffman's claim apparently uncritically. I have to confess to being extremely skeptical that clinical notes based on recollections gathered some time after a 45-minute session will indeed provide an account of the proceedings that remains as untainted by unconscious motivation as Hoffman anticipates.

Irrespective of the epistemic status of clinical reports, it is beyond doubt that the experience of engaging in clinical work offers most of us a far more direct and important route to progress in treatment techniques than the most sophisticated process-outcome studies. Speaking personally, it was the repeated failures I experienced working with severely personality disordered patients using classical analytic methods that led me to explore non-insight-oriented, mentalization-based techniques and attachment theory conceptualizations (Fonagy & Target, 2007). This brings us to the second substantive point, the status of the case study.

## THE PSYCHOANALYTIC CASE STUDY

The second controversial issue identified by Safran concerns the privileging of quantitative research over the case study method. As Safran pointed out, stating these as epistemic alternatives is a mere rhetorical device to facilitate the contrast of “constructivism” with “objectivism.” We can all see that the dichotomy is falsely stated. No self-respecting scientist would deny the role of meaning-making in interpreting findings. We all know that in an ideal world facts should speak for themselves but sadly in our human scientific world they often appear to do so only when accompanied by a chorus of approval. This admission does not indicate epistemic equivalence between research and clinical pursuits. At issue here is not only the appropriateness or otherwise of single subject methodology for scientific advance in general but the particular appropriateness of such methods for psychoanalysis. Let me summarize briefly the argument against the psychoanalytic case report as advanced by Lewis Wolpert and then consider a defense.

Wolpert's argument was not against case reports per se. He accepted the importance of case studies in medicine in general and psychiatry in particular. However, he rejected the validity of reports of individual treatments that are not presented against a backdrop of a tightly knit web of scientific theories concerning psychopathology and the theory of therapeutic effects. Wolpert's claim is that in the absence of a coherent and robust understanding, there is “nothing useful to be learned from a case report on an individual that could help other patients. Each case is essentially the relationship between an analyst and the patient” (Wolpert & Fonagy, 2009, p. 483). On reading Hoffman's article I would presume that he would concur with Wolpert. But whereas Hoffman feels that such uniqueness should be a spur to the further exploration of human idiosyncrasy, Wolpert feels that the strength of individual case reports lies in detailed observation being able to disconfirm previously held preconceptions. Studied in adequate detail and with sufficient regard for controlling for obvious alternative accounts, a single case can present a conjunction of phenomena that might require a radical reevaluation of prior beliefs.

But a coherent theoretical frame is essential to this process. Repeated demonstrations of individuality and uniqueness, and a general lack of predictability, however emotionally and intellectually gripping, will mostly fail for scientific purposes and will be of limited epistemic value other than the didactic function of illustrating the author's strongly held convictions. Studying the history of psychoanalysis, it is hard to disagree with Wolpert. Wolpert cited Arnie Cooper's (2008, p. 235) trenchant critique of contemporary pluralism, which “is to a surprising degree a multiplicity of authoritarian orthodoxies, each derived from a particular thinker rather than a scientific discourse.” A semi-inevitable consequence or indication of the absence of dialectical constructivism in modern psychoanalytic discourse is the increasing tendency on the part of psychoanalytic writers to cite a more and more narrow range of recent articles, rarely venturing outside their conceptual comfort zone (Fonagy, 2003).

If we are to retain psychoanalytic case reports in a mainstream psychiatric journal, we have to concede that special pleading for a unique epistemology of psychoanalysis is no more justified than it would be for any other subdiscipline of mental health sciences. Claiming that our science is governed by distinct epistemic principles is hard to support given the variety of alternative psychological frameworks attempting to capture the complexity of human subjectivity which are vying for recognition in psychiatry, each with different epistemic strengths and weaknesses. All are faced with similar challenges of sometimes elusive phenomena, the unsatisfactory nature of probabilistic inference, the need for special conditions for observation, and the risk that the available tools do not capture adequately the phenomena of greatest interest. However, we can assert that regardless of the standards of rigor of current clinical practice, there is nothing inherently unscientific about the psychoanalytic clinical situation. Further, the intensity of the interpersonal encounter involved in three or four times weekly psychotherapy has the potential to offer insights that are likely to be vital for a comprehensive appreciation of the human social condition. In particular, psychoanalytic observation enables us to question that which we might otherwise most readily overlook. I recall with appreciation Jonathan Lear's (1998) comment, “Psychoanalysis begins in wonder that the unintelligibility of the events that surround one do not cause more wonder” (p. 28).

The argument for case studies rests, in my view, in the potential for us to be blinded in ordinary discourse or observation to the proliferation of meanings that characterize the activity of human subjectivity. These all too readily escape what Lear (1998) termed “knowingness.” Our unique commitment as psychoanalysts is to relentlessly pursue and study the truth behind the ordinary, what Hannah Arendt (2006) evocatively described as “the banality of evil.” Human (self-)destructiveness, perhaps entailed in the classical metaphor of the death instinct (Freud, 1920), is hardly the preserve of fanatics or sociopaths. It is also characteristic of ordinary minds too willing to accept the premises of their social context, who persist in viewing their experience of their actions as normal. These ordinary encounters provide the nomological network of meanings that justify the description of psychoanalytic therapeutic practice even in high-impact scientific journals. The meanings that are created in the intersubjective space between patient and therapist are essential to the understanding of the complex relationship representations, where answers to the most fundamental questions about the human motivational system lie.

Hoffman offers the case of Neil, an impulsive man who was sent to a pharmacologist by his analyst to finally deal with his angry emotional outbursts. The condescending attitude of the physician enraged Neil so much that he vowed to control his temper on his own. He brought his consciousness to bear on his actions, and his self-report indicates a 98% reduction in the frequency of explosions. I am mentioning this example because on reading it I felt it was a wonderful illustration of the value of the psychoanalytic case report. It shows that, contrary to many psychoanalytic theories, insight could not be considered to provide a satisfactory account of Neil's dramatic improvement. One may speculate that the unconscious fear of his analyst's rejection (triggered by the referral to another professional) sensitized Neil to the attitude of the condescending physician. The rage with the doctor may also be seen as a displacement of anger with his analyst and his improvement as a transferentially provoked flight into health (Dysart, 1977; Wallerstein, 2000). But the dynamic unconscious was largely silent in this intriguing incident.

What Hoffman's tiny example powerfully points to is the dramatic and radical need for a psychoanalytically clinically informed reconsideration of the nature of consciousness, which might ultimately shed some light also on the dynamic unconscious and the relations of the conscious and unconscious minds. The focus of psychoanalytic interest has always been the unconscious. In his “Outline of Psychoanalysis,” Freud (1938) wrote, “There is no need to characterize what we call ‘conscious’: it is the same as the consciousness of philosophers and of everyday opinion” (p. 159). Consciousness is taken as given and the contents of consciousness are viewed merely as signposts to the depths. Present preoccupations, memories, current or recent physical sensations and descriptions, physical manifestations of emotions, and more are explored in pursuit of associative networks that give hints of the mental states hidden and disguised by consciousness. This principle is one of the best established tenets of our discipline. But Neil's example suggests that seeing consciousness merely as a route to concerns outside awareness underestimates its role in the dynamics of mind and clinical psychoanalysis. In the “Outline” Freud himself did acknowledge the mysteriousness of consciousness, stating that “the starting-point for this investigation is provided by a fact without parallel, which defies all explanation or description—the fact of consciousness” (p. 157). It is self-evident that the unconscious mind cannot be explicated without reference to consciousness.

## THE NATURE OF DOUBLETHINK AND THE NATURE OF ACADEMIC DISCOURSE

It is my sense then that Irwin Hoffman, Jeremy Safran, and I are broadly in agreement about the unique value of psychoanalytic case methods. Nor would I disagree that adequate critical scrutiny of clinical material can advance the inquiry of the theoretician as well as the skills of the clinical analyst. My own reporting of cases has, in one instance at least, benefited from Hoffman's critical commentary (Hoffman, 2004). So, given the deep level of agreement between these three serious scholars (forgive the presumption in considering us to share this distinction), what may be the motivation behind setting up an opposition between an idealized clinical analyst and a doublethinking Orwellian researcher? Given the choice, most of us would wish to think of ourselves as a person who “will stand for human freedom, for the dignity of the individual, for the meaningfulness of community, and for the sacrosanct integrity of every moment of experience” (Hoffman, 2009, pp. 1064–1065). Equally, few would opt to be psychoanalytic researchers if this inevitably implied engaging in “subtle sado-masochistic complicity.” More puzzling for both Safran and me is the assertion that researchers want to acquire and maintain “dominance over other conceptions of psychoanalysis and other views as to what psychoanalysis *…* requires to establish its legitimacy” (p. 1058). I have to say that, as someone who particularly values saying things explicitly, even when these ideas may prove to be unpopular, who came to value psychoanalysis precisely because it was committed to pursuing that which one was most motivated to avoid, I find it a little harsh to be depicted (even by implication) as someone who engages in “conscious deception while retaining a firmness of purpose that goes with complete honesty” (Orwell, Atet 1949*/*1961, p. 176).

However, all this is part of the rough-and-tumble of academic debate. It has always been thus. Finding common ground is far less exciting than creating imaginary philosophical disparity. Safran's concern is to assert the importance of public demonstration of effectiveness both for policymakers and clients of psychoanalytic therapy, many of whom, in seeking treatment, wish for no more than relief from psychic pain. I naturally fully support this position, but wish to add a further important research priority for which allowance must be made by clinicians. My concern here is not primarily to do with Hoffman's opposition to research as fact finding, but what Safran also eloquently points to in his response: the misrepresentation of realistic acceptance of the limitations of one's method as some deep bizarrely motivated strategy to disempower one's opponents by the cunning device of humility. In my view, the attribution of capricious motive to what is simple scientific principled honesty is deeply destructive, unscientific, and verging on the immoral. Without acknowledging the limitations of our research methods, without exercising constant and searching critique of the limitations of our findings, there can be no place for us at the table of 21st–century academe.

Psychoanalytic researchers are accustomed to unprovoked and uncalled for attacks on their work both from psychoanalytic colleagues and from academics hostile to analytic ideas. The researchers’ interests have to be sustained by the subject matter, as the intrinsic rewards, in terms of either academic promotion or popular recognition (by clinicians), are scarce. Most have an ideological commitment and a concomitant wish to preserve psychoanalysis for university education where most of us first encountered psychoanalysis. Consciously or unconsciously, rightly or wrongly, I believe researchers sense that unless they can keep the flame of Freudian thinking alive within universities the entire enterprise is doomed. The recruitment of bright energetic young people is the lifeblood of every specialty. Researchers have to survive in increasingly harsh academic environments and have to intrigue students sufficiently for them to pursue psychoanalytic interests. To ensure that psychoanalytic psychology will be taught to the next generation there is no alternative but to engage with 21st-century academic discourse. Like it or not, in most academic centers this is the discourse of neuroscience and large sample size research. Psychoanalytic researchers do not wish to constrain the clinician's right to place at the forefront of their scientific concern, the individuality of the patient and centrality of the person of the therapist. In fact, it is precisely this kind of “first-person psychology” (Northoff & Heinzel, 2006) that they wish to explicate in their work.

## CONCLUSION

So why use such strong rhetorical language to distance the clinical community from the individuals who try and protect the right of patients to access psychoanalytic therapies and the opportunity of students to take an interest in psychodynamic psychology and clinical work? Given that neither Jeremy Safran nor I see substantive differences between the value system that Hoffman's paper lays claim to and our own research-grounded moral and epistemic stance, I can see little merit in either engaging in debate or rebutting tendentious moralizing rhetoric.

What Hoffman calls doublethink is nothing but the recognition by all self-respecting scientists, including both Safran and myself, of the limitations of scientific methodology. At any one time scientists make do with the best they have. They hope to make progress with as much awareness of the limitations of their methodology as possible. I hope that Hoffman's call for a genuinely critical dialogue in relation to the case study method integrates as much of this “doublethink” as he can tolerate.
